# Comparison of the calcium-related factors in Parkinson’s disease patients with healthy individuals

**DOI:** 10.22088/cjim.11.1.28

**Published:** 2020

**Authors:** Sadra Samavarchi Tehrani, Mohammad Sarfi, Tooba Yousefi, Alijan Ahmadi Ahangar, Hemmat Gholinia, Reza Mohseni Ahangar, Mahmoud Maniati, Payam Saadat

**Affiliations:** 1 Department of Clinical Biochemistry, School of Medicine, Tehran University of Medical Sciences, Tehran, Iran; 2 Student Scientific Research Center, Tehran University of Medical Sciences, Tehran, Iran; 3 Student Research Committee, Babol University of Medical Sciences, Babol, Iran; 4 Mobility Impairment Research Center, Health Research Institute, Babol University of Medical Sciences, Babol, Iran; 5 Health Research Institute, Babol University of Medical Scences, Babol, Iran; 6 School of Medicine, Ahvaz Jundishapur University of Medical Sciences, Ahvaz, Iran

**Keywords:** Parkinson’s disease (PD), Calcium-related factors, Biochemical markers

## Abstract

**Background:**

Parkinson’s disease (PD) is one of the most common neurodegenerative diseases (ND). Studies have demonstrated that biochemical markers have an association with PD. We aimed to investigate an association of biochemical markers including calcium, vitamin D, alkaline phosphatase (ALP), parathormone (PTH), and phosphorous with PD.

**Methods:**

This study was conducted on 139 PD patients and 100 healthy individuals. Serum levels of calcium, phosphorous, ALP, PTH and vitamin D were evaluated. Furthermore, student’s t-test and logistic regression models were used by SPSS.

**Results:**

The mean levels of calcium (9.4±0.7 and 9.0±0.8 ) and vitamin D (29.7±22.1 and 25.8±23.7) were higher in PD patients as compared with healthy controls, which only status of calcium being significantly different in the two groups (P<0.001). Levels of ALP (202.4±96.7 and 242.9±142.4) and phosphorous (3.6±0.6 and 4.22±1.1) were significantly different comparing PD patients with healthy subjects (P<0.01, P<0.001, respectively). ALP and phosphorous were significantly different in the two groups (OR=0.996, [CI 95%, 0.994-0.999], P<0.001, OR=0.475, [CI 95%, 0.325-0.694], P<0.001, respectively). Furthermore, increased levels of calcium resulted in an elevated risk of PD (OR=2.175, [CI 95% 1.377-3.435], P<0.001).

**Conclusion:**

Results show that mean levels of calcium are higher in PD patients relative to healthy controls. Thereby, higher levels of calcium may be associated with PD.

Parkinson’s disease (PD) is the second most common neurodegenerative disorder after Alzheimer’s disease (1). Increasing data have shown that the prevalence of PD is rising from about 0.6% in 65-69 year-olds to approximately 3.5% in 85-89 year-olds (2, 3). Regarding the increased population of the elderly, it seems that with increased life expectancy the incidence of PD rises considerably worldwide (4). Clinically, PD is characterized by alterations in motor function including tremor, rigidity, bradykinesia, rigidity, and postural instability, which are known as the cardinal features of PD. Non-motor manifestations such as anosmia, cognitive decline, personality changes, depression, anxiety, fatigue, dysautonomic symptoms, and some others are also present in the disease due to pathophysiological changes in the disease (5, 6). The clinical manifestations of PD are based on degeneration and loss of dopaminergic neurons in substantia nigra pars compacta (SNc) dopamine neurons (7). Besides, several pathophysiological processes of PD have been found to contribute to neuronal lesions such as mitochondrial and lysosomal dysfunction, dystrophic neurites, oxidative damage, protein aggregation, inflammation, and the presence of eosinophilic intracytoplasmic Lewy bodies (8, 9). Furthermore, it has been indicated that both the environmental and genetic factors are presumably involved in the development of PD (10). 

Recently, many studies have demonstrated that different biochemical markers are associated with the pathogenesis of PD ( 11 , 12 ). One of the most important factors associated with PD is calcium, which has a critical role in the function of neurons, is involved in the transmission of the depolarizing signal and synaptic activity (9). In addition, several reports have revealed that calcium dysregulation is involved in the pathogenesis of PD (13-15). Another biochemical marker which can affect the progression symptoms of PD is p*arathyroid hormone* (PTH). Besides, it seems that parathyroid gland function is impressible in the severity of symptoms of PD (16). 

Besides, many studies have shown the association of biochemical markers including alkaline phosphatase (ALP), vitamin D, and phosphorous with PD (17, 18). They reported that serum levels of bone-ALP significantly increase in PD patients to compare the healthy controls, and found that the difference of serum status of phosphorus was not significant between the two groups (17). Furthermore, PD might be caused by an unceasingly inadequate vitamin D levels leading to a chronic loss of dopaminergic neurons in the brain (19). Epidemiologic studies have also proposed an inverse association between serum levels of vitamin D and risk of PD (20-22). 

Moreover, given the paucity of research on serum levels of biochemical markers in PD and the existing doubtful reports about the association between biochemical markers with PD on the one hand, and the emotional problems, posed by PD for patients and their families, along with huge financial expenses imposed upon families and health authorities for the care and maintenance of these patients especially in the late stages of disease, the current study was aimed to evaluate the associations of serum statues of important biochemical markers including calcium, phosphorus, ALP, vitamin D, and PTH with PD.

## Methods

***Study population: ***This study was conducted on 139 patients diagnosed with PD and 100 healthy individuals being similar to the patients in terms of age and gender. The participants of the healthy group were selected from healthy individuals who only performed routine checkups and lab tests at clinical laboratory and had no neurological diseases. The mean and standard deviation (SD) of the patients’ and healthy individuals’ age was (68.78±10.70) and (68.45±11.47), respectively. Informed consent was obtained from all subjects and the study was approved by the Ethics Committee of the Babol University of Medical Sciences (9603414- 4405). 

The inclusion criterion of this study was being diagnosed with PD. The diagnosis of PD was based on the four classic cardinal symptoms of the disease, namely tremor, rigidity, bradykinesia, and postural instability. Diagnosis of PD was based on the movement disorder society (MDS) clinical diagnostic criteria for PD, under the supervision of a neurologist who led the study (23).

**Exclusion criteria:** Excluded from this study were those who previously had a PD history and were under medical treatment. In addition, patients with Parkinson's syndromes that consumed neuroleptics or had Parkinson's syndromes with other known causes that are not a primary idiopathic PD were excluded. Finally, patients were excluded if they had other known causes of osteoporosis, renal and hepatic impairment, or a history of therapy with calcitonin, calcium, vitamin B, vitamin D, and folic acid. Obviously, patients who did not cooperate were also excluded. 

**Laboratory assessment:** In both patient and healthy groups, Calcium (Ca), Parathormone (PTH), Alkaline Phosphatase (ALP), vitamin D levels and phosphorus (P) were measured in Laboratory of Ayatollah Rouhani Hospital. Calcium (normal range: 8.2-10.6 mg/dL) was performed by photometric UV test, and P (normal range: 2.5-4.5 mg/dL) by photometric with ARSENAZO III methods. ALP (normal range: 64‑306 mg/dL) was performed by spectrophotometric methods (Hitachi 902autoanalyzer) with pars azmoon kit. PTH (normal range: 10‑65 IU/L) and vitamin D were analyzed by enzyme immunoassay by Pars Azmoon kit according to the protocol. PTH with Euroimmun microplate ELISA and vitamin D with 25-oH vitamin D ratio diagnostic kit

**Statistical analysis:** In this study, statistical analyses were performed by SPSS Version. 24.0 (SPSS, Chicago, IL). The mean values±standard deviation (SD) in the PD patients and healthy individuals were compared using student’s t-test. Furthermore, associations between PD and biochemical markers such as vitamin D, PTH, ALP, calcium, and phosphorous were analyzed by logistic regression models. In addition, the diagnostic accuracies of the calcium and calcium-related factors for discrimination of healthy controls and PD patients were analyzed by receiver operating characteristic curves (ROC). Data were presented as mean ±SD. Finally, p<0.05 were considered statistically significant.

## Results

The study included 139 patients with PD, and 100 healthy individuals. The PD patients were 76 males and 63 females (mean±SD age 68.78±10.70 years), and the healthy subjects included 47 males and 53 females (mean±SD age 68.45±11.47 years). The biochemical measurements are shown in table 1. The results indicate that the mean serum levels of calcium and vitamin D were higher in PD patients relative to healthy controls. We observed that only the serum status of calcium was significantly different comparing PD patients with healthy controls (p<0.001). Besides, the results of the present study also revealed that serum levels of PTH, ALP, and phosphorous were lower in PD patients as compared with healthy individuals, with only the levels of ALP and phosphorous being significantly different between two groups (p<0.01, p<0.001, respectively). The logistic regression model indicated that among the biochemical markers, only phosphorous is significantly different in the PD patients and healthy subjects (odds ratio 0.24, [CI 95%, 0.118-0.419], p<0.001,) (table 2). To examine the diagnostic accuracy of calcium and calcium-related factors for distinction between normal individuals, and the PD groups, ROC analysis was performed and the results were presented in table III. Our results showed that for the discrimination between the PD groups and the healthy subjects, calcium, ALP, vitamin D, and phosphorous had a reasonable AUC

**Table 1:** Serum levels of calcium and calcium-related factors in the study population

**Parameters**	**PD patients** **N=139** **Mean± SD**	**Healthy individuals** **N=100** **Mean ± SD**	**P-value**
Age	68.78±10.70	68.45±11.47	0.81
Vitamin D (ng/ml)	29.78±22.17	25.85±23.73	0.20
PTH (IU/L)	45.23±24.26	51.68±37.11	0.11
ALP (mg/dl)	202.43±96.78	242.96±142.45	0.01
P (mg/dl)	3.65 ± 0.6544	4.22±1.12	<0.001
Ca (mg/dl)	9.40 ± 0.74	9.02 ± 0.83	<0.001

Values are given as Mean ± Standard Deviation (SD)

Abbreviation: PD (Parkinson’s disease), PTH (Parathyroid hormone), ALP (Alkaline phosphates), P (phosphorous), Ca (calcium)

**Table 2:** The risk of Parkinson’s disease with adjusted variables by logistic regression test

**Parameters**		**Odds Ratio(OR)**	**CI 95%**	**P.value**
**Sex**	Male	1	-	-
	Female	1.088	0.581-2.037	0.793
Age (Year)	40-60	1	-	-
	61-80	1.197	0.630-2.276	0.583
	81-100	0.786	0.317-1.948	0.603
Vitamin D (ng/mL)	<30	1	-	-
	≥30	0.767	0.429-1.372	0.115
PTH (IU/L)	<10	0.229	0.045-1.166	0.07
	10-65	1	-	-
	>65	0.601	0.310-1.162	0.130
ALP (mg/dl)	<62	1.118	0.183-6.842	0.904
	62-300	1	-	-
	>300	0.516	0.211-1.263	0.148
P (mg/dl)	<2.5	0.593	0.036-9.639	0.713
	2.5-4.5	1	-	-
	>4.5	0.241	0.118-0.419	<0.001
Ca (mg/dl)	<8.5	2.047	0.532-7.884	0.291
	8.5-10.5	1	-	-
	>10.5	0.481	0.107-2.162	0.340

**Table 3:** Comparison of diagnostic accuracies of biochemical parameters by ROC analysis for discrimination of the healthy persons than the Parkinson disease patients

**Variable **	**AUC** **(CI 95%)**	**P-value**
Calcium (mg/dl)	0.64 (0.57-0.72)	0.001
Phosphorous (mg/dl)	0.66 (0.58-0.73)	0.001
ALP (mg/dl)	0.62 (0.53-0.68)	0.007
Vitamin D (ng/mL)	0.61 (0.52-0.67)	0.010

## Discussion

We assessed the serum level of biochemical markers such as calcium, PTH, ALP, and phosphorous, and vitamin D in PD patients and in age- and sex-matched healthy individuals. We examined the relationship between these factors and calcium in PD patients in comparison with healthy subjects. The results of our study indicated that there was a significant difference between the two groups in terms of the serum levels of phosphorous. In addition, decreased levels of phosphorous resulted in an elevated risk of PD. 

PD is a multifactorial ND with diverse molecular and neural disturbances such as autophagy and mitochondrial disruption, ER stress and deregulation of calcium hemostasis which trigger Dopaminergic (DA) neural death in the Substansa Nigra (SNs) located in the midbrain (24). Loss of calcium hemostasis causes neural cell death in SNs. Abnormal calcium hemostasis in DA neurons might be due to the consequences of mitophagy disruption, ER stress, mitochondrial dysfunction, and α-synuclein aggregation. DA neuron survival has been shown to reduce in the situation with upper or lower level of calcium (25). Multiple molecular disorders can affect the calcium equilibrium in DA neurons. For instance, over-activity of NMDA receptors (26), malfunction of voltage-dependent calcium channels, and genetic mutation in DJ-1 gene, which has a protective role against oxidative stress (27). High levels of calcium stimulate excessive dopamine biosynthesis, which causes autointoxication in DA neurons (28, 29). Moreover, calcium overload in neurons leads to mitochondrial dysfunction, induction of oxidative stress, and reduced bioenergetics capacity (30, 31). 

Therefore, high levels of calcium have a direct correlation with PD, which arises from the disruption in calcium hemostasis factors including vitamin D, PTH and ALP. Many studies have demonstrated that these factors are associated with calcium disruption in PD. Sato et al. for example, compared the level of 25-OH vitamin-D, PTH and bone mineral density (BMD) in a group of stooped posture, a hallmark of PD, with a group of non-stooped posture. According to their results, 25-OH vitamin-D and BMD were lower, while PTH was higher in stooped posture (32). In their previous study, they had already shown that the low level of vitamin D was due to the bad nutrition and reduced exposure to sunlight (33), which arise from inability to move properly. In another study, the serum levels of vitamin D_2_ and vitamin D_3_ were investigated in a large number of PD patients. It was shown that there is an inverse relationship between the level of vitamin D and PD. Moreover, no difference was found between vitamin D_2_ and D_3 (_34_)_. Therefore, it can be concluded that vitamin D can be considered as a protective factor against PD. Meanwhile, to investigate the correlation between vitamin D_3_ deficiency/insufficiency and PD, Fullard et al found that there was no significant difference in vitamin D between the plasma of high-risk PD compared to that of other matched groups. Their study shows no relationship between the low level of vitamin D and DA neural cell death (35). Vitamin D and calcium have an inverse relationship with PTH, meaning that when there is a low level of vitamin D and calcium, the parathyroid gland is stimulated to increase the secretion of PTH (36). Moreover, low levels of vitamin D can have a negative effect on calcium absorption, which indirectly stimulates the biosynthesis and secretion of PTH to compensate for calcium insufficiency. On the other hand, high level of PTH can directly affect bone resorption and absorption and finally lead to bone fractures (32, 37). The bone abnormality in these patients might arise from the high level of PTH which leads to poor posture in PD patients. In another study performed by Sato et al., the high level of calcium (38) was found to inhibit PTH secretion, thus, they concluded that PTH was lower in PD patients, in comparison to normal controls (21). Although in another study by the same researchers on the patients with stooped posture, the PTH level was reported to be higher in PD patients with stooped posture as opposed to PD patients with no stooped posture. 

In this study, our results showed that calcium and vitamin D levels were higher in PD patients compared with normal controls, while PTH level was lower in these patients, which is in line with the effect of vitamin D and calcium on PTH secretion. In addition, the current study evaluated the plasma levels of ALP and phosphorous, both of which were lower in PD patients. The increased levels of ALP and phosphorous did not correlate to PD directly, but calcium level had a direct correlation with PD. Our results also showed that lower levels of phosphorous increase the risk of PD, and this decreased level of phosphorous might be due to the primary increase of PTH hormone in PD (39). Besides, the serum level of phosphorus and ALP was significantly different between two groups, which can be related to calcium abnormality in PD. However, further large-scale studies are needed to verify this hypothesis, and there are still some potential limitations that merit considerations. These include low sample size, difficult accessibility of the patients to the staff, loss of some patients and financial issues. 

In summary, we found that only serum levels of calcium were significantly different comparing PD patients with healthy control. Besides, we observed that only serum levels of ALP and phosphorous were significantly different in the two groups. Among the biochemical markers detected, only levels of calcium did induce more chance for PD, the so increased level of calcium results in elevation of risk of PD. 
